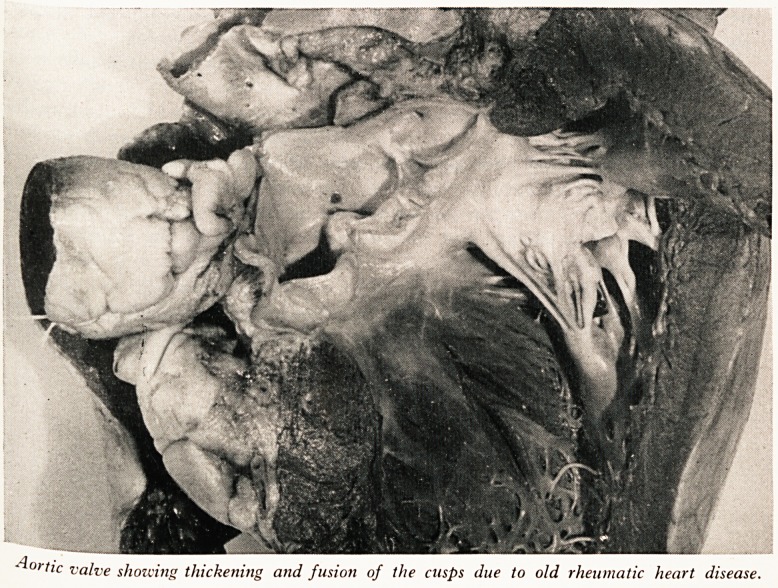# Maternal Death Due to Rheumatic Heart Disease

**Published:** 1956-04

**Authors:** T. F. Hewer


### MATERNAL DEATH DUE TO RHEUMATIC HEART DISEASE

*A Clinical Pathological Conference of the University of Bristol Medical School*

CHAIRMAN: PROFESSOR T. F. HEWER

*Mr. A. Fyfe:* The story begins in 1947, when this lady was 20 years old and found to have pulmonary tuberculosis; there was no history of rheumatic fever. In 1949 she spent 10 months in a sanatorium. In April 1952 she was admitted to Southmead Hospital three months pregnant; on account of the activity of the tuberculosis her pregnancy was terminated. At this time it was noted that she had signs of mitral stenosis and aortic incompetence. Her condition improved and when she became pregnant again, in February 1954, she was seen by Dr. Roberts who considered her chest lesion was quite satisfactory. Her confinement was due on 13th November 1954. In July she was sent to see Professor Perry on account of dyspnoea. He found she had chronic rheumatic heart disease with fully developed mitral stenosis and considerable fibrosis in the left lung following tuberculosis. Her pregnancy progressed satisfactorily and she was seen by an obstetrician who arranged that she should be admitted to hospital on the 5th November in preparation for delivery. She was admitted in labour on the 5th November. She was not dyspnoeic, and her pulse rate was 110. During the course of that night it went up to 120 but it settled down to 96 by the morning. After 22 hours in the first stage she was fully dilated and had a forceps delivery under local anaesthesia. The second stage lasted half an hour: it was quite a straightforward forceps delivery and it was not until afterwards that she showed any signs of marked dyspnoea or cyanosis. Dr. Wright, who was present at the delivery, can continue the story of her subsequent cardiac condition.

*Professor Lennon:* Was she admitted at the 28th week for rest and assessment?

*Mr. Fyfe:* No; she was seen up to 22 weeks. There is a note to the effect that she was to see Professor Perry but no record that she actually did so.

*Professor C. B. Perry:* We saw her in July of last year and we understood that the chest physicians were happy for the pregnancy to continue; she was then 4½ months pregnant and a little dyspnoeic on hills. We thought she would be all right. She was seen again on the 21st October when she said she was fit; her breathlessness was less and the physical signs were unchanged: we asked her to see us after the baby was born.

*Question:* What analgesia was used?

*Mr. Fyfe:* Trilene.

*Professor Perry:* When she came in, in labour, there was no evidence of congestive failure: is that right? (Mr. Fyfe said it was.)

*Dr. Wright:* I first saw this lady some time after she had been in labour, when the os was about three-quarters dilated. We questioned her about the reason why she did not come in two weeks before delivery and she said she had been better and rather less breathless. She said she could manage to get plenty of rest at home. During labour she was not unduly distressed; her breathing was fairly good. She had the clinical signs of mitral stenosis and aortic incompetence. In the chest there was less movement on the left than the right; there were no adventitous sounds at the bases. Labour was progressing quite well and we said it would be better to have a forceps delivery in view of the cardiac and pulmonary lesions. She seemed quite comfortable in the second stage but as she was being delivered her colour changed; she became cyanosed, with coughing and pulmonary oedema. After a while her colour improved and she was able to talk to her husband and saw the baby. For the next two or three hours her breathing was easier and her colour better and things seemed to be going all right. About $3\frac{1}{2}$ hours later her temperature went up to 104°, the pulse became rapid and irregular, and she developed a slight cough. In the early morning she had another attack of pulmonary oedema. About 4 a.m. her condition deteriorated; the blood pressure fell and she died at 12.10 p.m. on the 6th November.

*Professor Perry:* What was the treatment?

*Dr. Wright:* She was given 20 c.c. of cardophyllin intravenously immediately the pulmonary oedema developed; she had some morphia gr. 1/6 and intravenous digoxin 0·5 mg. in normal saline. She responded for a while and was given oxygen by mask and then by nasal catheter afterwards. She was given two further doses of 1/6 grain of morphia during the night, and, in view of the possible lung infection, some penicillin which was later changed to aureomycin.

*Dr. J. M. Naish:* I saw this patient in the morning about 9.30 a.m. and there were questions which I could not answer: why had she had the high temperature? Why had she such a degree of peripheral circulatory failure? Her lungs were then temporarily free of oedema and I questioned whether she might have had a pulmonary infarct, complicating the pregnancy and rheumatic heart disease. I wondered whether there was perhaps some infective complication causing the rapid pulse or whether she had paroxysmal tachycardia. I suggested some measures to get the systolic blood pressure up—it had dropped to 60 mm. Hg. A noradrenaline drip was set up but she died before it could have any effect on her condition. She is a mystery to me because of the high temperature, the peripheral circulatory failure coming a long time after delivery and the tremendous tachycardia. We have looked up records of cardiac failure during and after delivery and there has been one death in the last few years of a patient with rheumatic heart disease who died of pulmonary oedema after delivery in much the same way, but she did not have adequate antenatal care. We have had two other cases of rheumatic heart disease, with severe pulmonary oedema after delivery, who recovered. We do see cases of acute pulmonary oedema and heart failure during delivery but not very often with rheumatic heart disease and in this case I do not know why she had a high temperature. When I saw her she was collapsed and shocked and I noted that she had some dullness in the left apex which was presumably due to old fibrosis but we wondered whether she had an infarct as well. Her blood pressure fell approximately 12 hours after delivery.

*Professor Perry:* It did not go up in between?

*Dr. Naish:* The blood pressure responded during the night reaching 120/80 and then she went into peripheral circulatory failure again.

Professor Hewer asked Dr. Brown to present the post-mortem findings.

*Dr. N. J. Brown:* There was slight oedema of the ankles, otherwise no external abnormality. There was a slight excess of fluid in all the serous cavities. The heart was enlarged and increased in weight. The mitral valve (Plate IV) showed considerable thickening and stenosis with fusion of the cusps and shortening and thickening of the chordae tendineae. The left ventricle was hypertrophied and dilated. The aortic valve was also thickened with fusion of the cusps and incompetence. There were similar but less severe rheumatic changes in the tricuspid valve. Histologically there was no evidence of any active rheumatism.

The right lung showed intense pulmonary oedema. The left lung weighed a quarter of the right and showed not so much oedema but it was contracted by old tuberculous scarring in the upper lobe. Histologically no tuberculous lesions were seen but bronchopneumonia was commencing in both lungs.

*Mitral valve showing scarring and fusion of the cusps and shortening and thickening of the chordae tendineae. There is also left ventricular hypertrophy.*

*Aortic valve showing thickening and fusion of the cusps due to old rheumatic heart disease.*

There was no evidence of chronic venous congestion in any organ. The uterus was in the normal puerperal state.

This was a case of severe chronic rheumatic heart disease, with pulmonary oedema, and commencing bronchopneumonia, but with no evidence of congestive heart failure.

*Question:* Can you say how old the bronchopneumonia was?

*Dr. Brown:* Probably 24 hours.

*Question:* Do you think this patient had such a very severe degree of pulmonary oedema because she only had one lung for expansion?

*Professor Perry:* I do not know; this case is very worrying. I have been looking at Bramwell's book again; out of 300-odd cases, 15 deaths were, he thought, attributable to a heart lesion and I cannot find a case like this at all: his cases had congestive failure before confinement. I suppose that during pregnancy there is a large arterio-venous shunt through the placenta and we know that the sudden closure of an arterio-venous shunt in a normal person may cause acute failure. Whether the fact that she only had one functioning lung made any difference I do not know. I should have thought perhaps venesection might have been of value.

*Professor G. G. Lennon:* I should like to stress the importance of seeing a patient at 28 weeks, even if she is very fit and obviously doing well. The two times when heart failure is most likely to occur are at the 28th week and at the 40th week.

*Question:* How long did she have peripheral circulatory failure?

*Dr. Naish:* She lived for three hours after the blood pressure began to drop. I tried to push up the peripheral blood pressure.

*Professor Lennon:* Regarding the use of trilene: this is a point which is going to be much more important in the future. When it is given for some time there may be undesirable complications. Some patients are sensitive to it. I wonder if it should be used in heart cases.

*Question:* I should like to ask about the diagnosis at the age of 20 of mitral stenosis and pulmonary tuberculosis. This combination is usually said to be very rare: must we therefore assume that she had pulmonary tuberculosis before the mitral stenosis?

*Professor Perry:* I think you can assume that she had the mitral stenosis long before the tuberculosis. The co-existence of the two is relatively rare considering how common each of the diseases is but it does occur.

We are still left in doubt as to the cause of the patient's death, partly because we do not yet know enough about the rapid circulatory adjustments that take place immediately after delivery. As Professor Lennon pointed out, there is some evidence that the blood volume and cardiac output reaches the two maxima at the 28th week of pregnancy and at or after confinement. However, this patient seems to have suffered severe peripheral circulatory failure and we can only presume that associated in some way with the pulmonary oedema she developed an acute pulmonary infection which gave rise to the fever and the circulatory collapse.

## Figures and Tables

**Figure f1:**
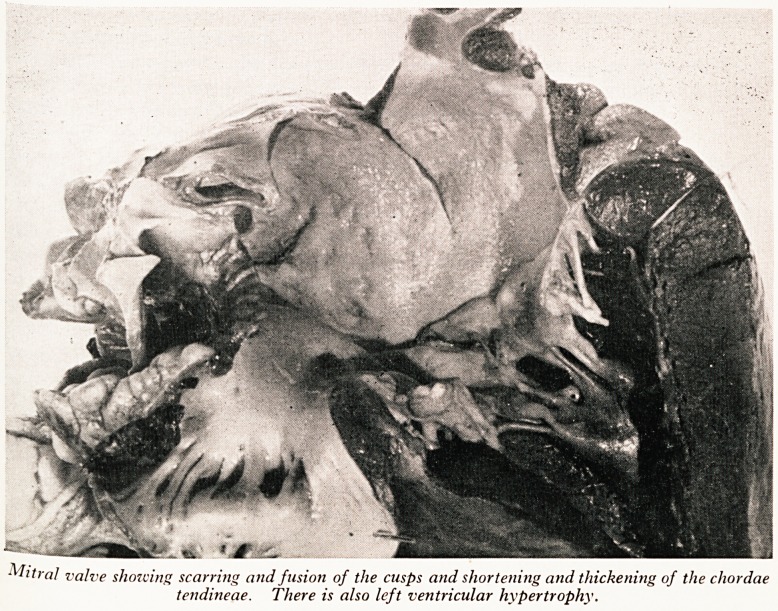


**Figure f2:**